# Far-lateral approach without C1 laminectomy for microsurgical treatment of vertebral artery and proximal posterior inferior cerebellar artery aneurysms: Experience from 48 patients

**DOI:** 10.1016/j.wnsx.2023.100216

**Published:** 2023-05-12

**Authors:** Kitiporn Sriamornrattanakul, Nasaeng Akharathammachote, Areeporn Chonhenchob, Atithep Mongkolratnan, Nattawut Niljianskul, I-sorn Phoominaonin, Chanon Ariyaprakai

**Affiliations:** Department of Surgery, Division of Neurosurgery, Faculty of Medicine Vajira Hospital, Navamindradhiraj University, Bangkok, Thailand

**Keywords:** Far-lateral approach, C1 laminectomy, Vertebral artery aneurysm, PICA aneurysm

## Abstract

**Background:**

In the endovascular era, most of vertebral artery (VA) and posterior inferior cerebellar artery (PICA) aneurysms were mainly treated with endovascular procedures. This study aimed to demonstrate the microsurgical treatment via the far-lateral approach without C1 laminectomy and its clinical outcomes.

**Methods:**

Forty-eight patients with VA and proximal PICA aneurysms treated by microsurgery through the far-lateral approach without C1 laminectomy, between January 2016 and June 2021, were retrospectively evaluated.

**Results:**

Most patients (87.5%) presented with subarachnoid hemorrhage. Grading at presentation was poor in 41.7%. The rates of VA dissecting aneurysms, saccular aneurysms of the VA–PICA junction, and true PICA saccular aneurysms were 54.2, 18.7, and 14.6%, respectively. All aneurysms were located above the lower margin of the foramen magnum. The far-lateral approach without C1 laminectomy was successfully used in all patients without residual aneurysms. Various surgical strategies were applied depending on the characteristics of the aneurysm. Good outcomes 3 months postoperatively were achieved in 77.1% and 89.3% for the overall and good-grade groups, respectively.

**Conclusions:**

Microsurgery is a safe and effective treatment of VA and proximal PICA aneurysms. Moreover, the far-lateral approach without C1 laminectomy was adequate and effective for aneurysms located above the lower border of the foramen magnum.

## 1 Introduction

Owing to the evolution of endovascular techniques and devices and the higher rates of microsurgical complications, vertebral artery (VA) and posterior inferior cerebellar artery (PICA) aneurysms are predominantly treated with endovascular procedures.^1^, ^2^, ^3^, ^4^, ^5^, ^6^, ^7^

Proximal PICA and VA aneurysms have a close relationship with the lower cranial nerves and brainstem and are reachable via the far-lateral or lateral suboccipital approach.^8^, ^9^, ^10^, ^11^ With the lower rates of aneurysm remnants and recurrences, the functional outcomes of microsurgery were favorable with endovascular treatment. However, the most important drawback of microsurgery was the higher rates of lower cranial nerve palsy.^1^^,^^7^ The relatively high surgical morbidity and mortality mainly resulted from the complicated surgical approach to the VA and proximal PICA, which are blocked by vital structures such as lower cranial nerves and brainstem.^1^^,^^12^

With well-developed microsurgical techniques and treatment strategies, microsurgery remains important in the treatment of VA and proximal PICA aneurysms with an acceptable rate of surgical complications and higher rates of aneurysm obliteration.^7^^,^^12^^,^^13^ The key surgical exposure for VA and proximal PICA aneurysms is the far-lateral approach, which has numerous variants. Matsushima et al suggested indications for drilling the condylar fossa and occipital condyle, which depends on the level of the lesion related to the hypoglossal canal, but the clear indication for C1 laminectomy was not established.^14^^,^^15^ This study aimed to demonstrate the surgical outcomes and reveal that C1 laminectomy is not necessary for VA and proximal PICA aneurysms in specific location.

## 2 Material and methods

We retrospectively reviewed data from patients who underwent microsurgery for the treatment of VA and proximal PICA (from the PICA origin to the caudal loop) aneurysms at the Faculty of Medicine Vajira Hospital, Navamindradhiraj University, between January 2016 and June 2021. We enrolled patients with ruptured and unruptured aneurysms. The main indications for open surgery were the financial aspect of endovascular devices. The exclusion criteria were as follows: distal PICA aneurysms (distal to the caudal loop), giant aneurysms, vertebrobasilar junction aneurysms, treatment with endovascular techniques, supportive treatment because of any problems, and follow-up loss. Data regarding the location of the aneurysms, aneurysm characteristics, surgical techniques, surgical outcomes, and Glasgow outcome score (GOS) at 3 months postoperatively were collected and analyzed.

### 2.1 Operative techniques

**Fig. 1:**
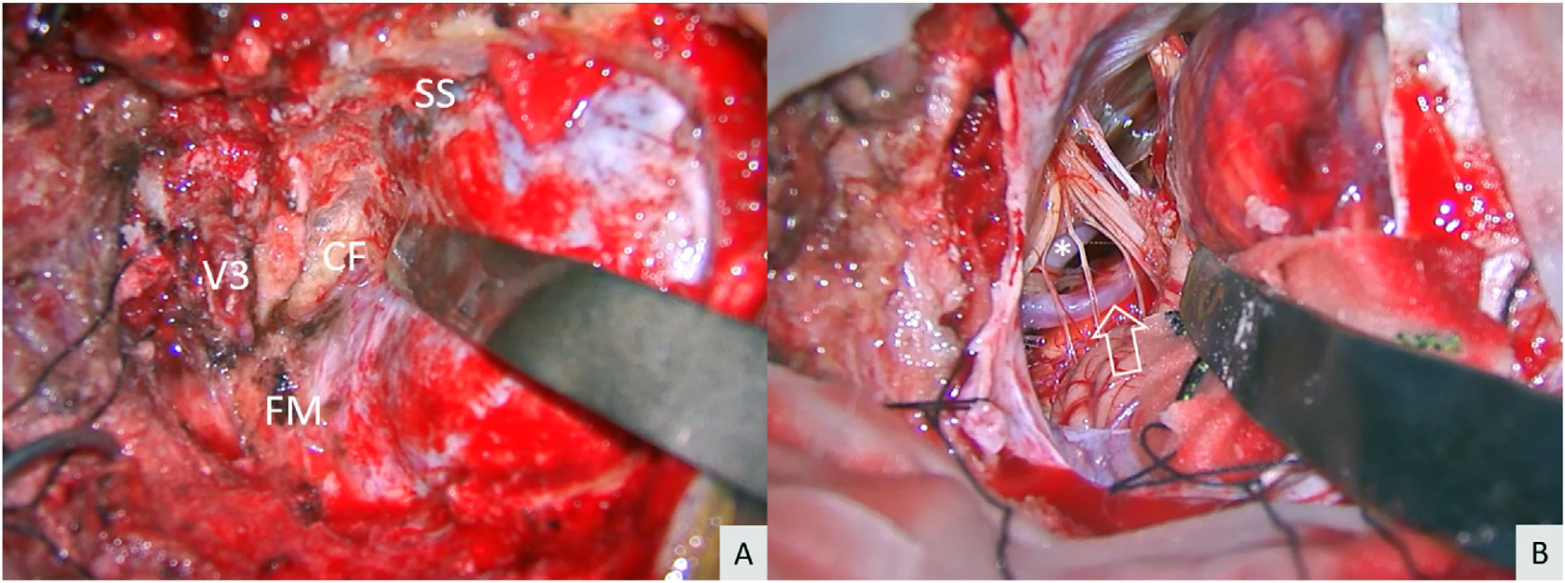
Left far-lateral transcondylar fossa approach (A) After the third segment of the vertebral artery (V3) and sigmoid sinus (SS) were exposed and the foramen magnum (FM) and condylar fossa (CF) were drilled, the transcondylar fossa approach was completed (B) After dural opening and elevation of the cerebellar tonsil, the lower cranial nerves, vertebral artery (asterisk), and posterior inferior cerebellar artery (arrow) were identified.

With the semiprone park-bench position, a paramedian linear or “L”-shaped skin incision was made. The occipital artery (OA) was harvested with an “L”-shaped skin incision in cases that need OA–PICA bypass. Suboccipital muscles were dissected in a layer-by-layer fashion. The V3 segment of the VA was exposed through the suboccipital triangle.^16^, ^17^, ^18^, ^19^ After retrosigmoid craniotomy was made, the posterolateral part of the foramen magnum was removed while preserving the posterior arch of C1. After the posterior condylar emissary vein was coagulated and cut, the condylar fossa was exposed, and the sigmoid magnum triangle, which is the posterior part of the jugular tubercle, was identified and drilled until the blue line of the hypoglossal canal was partially seen. The posterior part of the jugular tubercle, which is located above the hypoglossal canal, was drilled as much as possible (Video 1). At this step, the transcondylar fossa approach was completed (Fig. 1A). This approach was sufficient for lesions located above the hypoglossal canal. If the lesion is located below the hypoglossal canal, the occipital condyle, which is located below the blue line, should be partially drilled (transcondylar approach).^20^ After the dura was opened, the ipsilateral cerebellar hemisphere and tonsils were retracted superomedially to expose the V4 segment of the VA, proximal PICA, and lower cranial nerves (Fig. 1B). If necessary, OA–PICA bypass was performed first.^18^^,^^21^ Different aneurysm clipping techniques (direct clipping, trapping, proximal occlusion, distal occlusion, or blind-alley formation) was performed depending on the aneurysm characteristics.^13^^,^^21^

### 2.2 Outcome assessment

Outcomes were evaluated using GOS 3 months postoperatively, obliteration of the aneurysm, and new neurological deficits. GOS of 4 and 5 were defined as good outcomes, whereas GOS of 1–3 were defined as poor outcomes.

## 3 Results

### 3.1 Patient characteristics and clinical presentation

The study included 48 patients with VA and proximal PICA aneurysms. The average age was 56 years. Female patients were twice as many as male patients. The most frequent presentations were headache and altered consciousness (41.7%). Most patients (87.5%) experienced subarachnoid hemorrhage (SAH) at the initial presentation. The grading at the initial presentation was poor (World Federation of Neurosurgical Societies [WFNS] grades 4 and 5) in 41.7% of the patients. Patients with unruptured aneurysms were included in the good-grade group. VA dissecting aneurysms were the most common types (54.2%), followed by saccular aneurysms of the VA–PICA junction (18.7%). By aneurysm characteristics, dissecting, saccular, and fusiform types were found in 28 (58.3%), 19 (39.6%), and 1 (2.1%) patient, respectively. By location, VA, VA–PICA junction, and true proximal PICA aneurysms were found in 30 (62.5%), 9 (18.75%), and 9 (18.75%) patients, respectively, and all aneurysms are located above the lower margin of the foramen magnum. VA dissecting aneurysms were classified as pre-PICA, PICA, post-PICA, and non-PICA types^13^ (Table 1).

**Table 1:** Patient characteristics and clinical presentation.

Data	Total (*n* = 48)
Female:Male	32:16
Mean age in years	56
Presentation	
Headache	15 (31.2%)
Alteration of consciousness	10 (20.8%)
Headache with AOC	20 (41.7%)
Seizure	2 (4.2%)
Hemiparesis	1 (2.1%)
Subarachnoid hemorrhage	
Yes	42 (87.5%)
No	6 (12.5%)
Grading at presentation	
Good grade	28 (58.3%)
Poor grade	20 (41.7%)
Type and location of aneurysms	
VA dissecting aneurysm	26 (54.2%)
Pre-PICA type	6
PICA type	8
Post-PICA type	8
Non-PICA type	4
VA saccular aneurysm	3 (6.2%)
VA fusiform aneurysm	1 (2.1%)
VA-PICA saccular aneurysm	9 (18.7%)
True PICA saccular aneurysm	7 (14.6%)
True proximal PICA dissecting aneurysm	2 (4.2%)

AOC, alteration of consciousness; PICA, posterior inferior cerebellar artery; VA, vertebral artery.

### 3.2 Treatment and outcomes

The transcondylar or transcondylar fossa approach without C1 laminectomy was performed in all patients, of which 46 patients went through the ipsilateral approaches and two patients with the contralateral approach. For saccular aneurysms of the VA, VA–PICA, and true PICA, direct clipping and OA–PICA bypass with clipping were performed in 18 and 1 patient, respectively. For VA dissecting aneurysms without PICA involvement, proximal occlusion, distal occlusion, and complete trapping were performed in 15, 1, and 2 patients, respectively. For VA dissecting aneurysms with PICA involvement, OA–PICA bypass was performed in all patients (*n* = 8). The blind-alley formation and complete trapping were used in 6 and 2 patients, respectively. In one patient, fusiform VA aneurysm was obliterated with proximal occlusion. Two patients with proximal PICA dissecting aneurysms were treated with OA–PICA with trapping (Table 2). The average operative times were 5 and 6 h for non-bypass and bypass cases, respectively. The average temporary clip times were 8 and 29 min for non-bypass and bypass cases, respectively.

**Table 2:** Aneurysm characteristics, surgical techniques and complete aneurysm obliteration.

Aneurysm characteristics	Surgical technique	Number of patients	Complete obliteration
**VA, VA-PICA and true PICA saccular aneurysms**	Clipping	18	18
	OA-PICA bypass with clipping	1	1
**VA dissecting aneurysm**	Proximal occlusion (V4)	14	14
	Proximal occlusion (V3)	1	1
	Distal occlusion (V4)	1	1
	Trapping	2	2
	OA-PICA bypass with blind-alley formation	6	6
	OA-PICA bypass with trapping	2	2
**VA fusiform aneurysm**	Proximal occlusion (V4)	1	1
**True PICA dissecting aneurysm**	OA-PICA bypass with trapping	2	2
	Total	48	48

OA, occipital artery; PICA, posterior inferior cerebellar artery; V3, third segment of the vertebral artery; V4, fourth segment of the vertebral artery.

Complete aneurysm obliteration was achieved in all patients. If OA–PICA bypass (*n* = 11) was performed, good bypass patency was accomplished in all cases. Three months postoperatively, good outcomes were achieved in 37 (77.1%) patients for the overall and 25 (89.3%) patients for the good-grade groups. Three patients in the good-grade group had poor surgical outcomes. One 80-year-old patient with ruptured VA–PICA aneurysm developed severe sepsis from pneumonia postoperatively and died of respiratory failure. A 52-year-old patient with ruptured VA–PICA aneurysm developed aspiration pneumonia postoperatively. Another 76-year-old patient with ruptured proximal true PICA aneurysm developed postoperative hydrocephalus and ventriculoperitoneal shunt malfunction. After shunt revision, GOS 4 was achieved 10 months postoperatively. All patients with VA dissecting aneurysms achieved good outcomes (Table 3). No postoperative neck pain was detected in all patients at the last follow-up.

**Table 3:** Presenting grade and clinical outcomes 3 months postoperatively.

	Good outcomes (GOS 4,5)	Poor outcomes (GOS 1–3)	Total
Good grade (WFNS 1–3 ​+ ​unruptured cases)	25 (89.3%)	3	28
Poor grade (WFNS 4,5)	12	8	20
Total	37 (77.1%)	11	48

GOS, Glasgow outcome score; WFNS, World Federation of Neurosurgical Societies.

A new onset of dysphagia was detected in 2 (8%) patients with good grades who suffered from ruptured right VA–PICA aneurysms treated with direct clipping and ruptured left VA dissecting aneurysms with PICA involvement treated with OA–PICA bypass and blind-alley formation. For the latter patient, the high position of the caudal loop of the PICA concealed in the cerebellomedullary fissure was detected intraoperatively. The lateral medullary segment of the PICA was used as the recipient artery for bypass after intentionally sacrificing two roots of the spinal accessory nerve to access this segment of the artery. In both patients, the dysphagia spontaneously improved over time and completely recovered within 6 months postoperatively.

## 4 Illustrative cases

### 4.1 Illustrative case 1

**Fig. 2:**
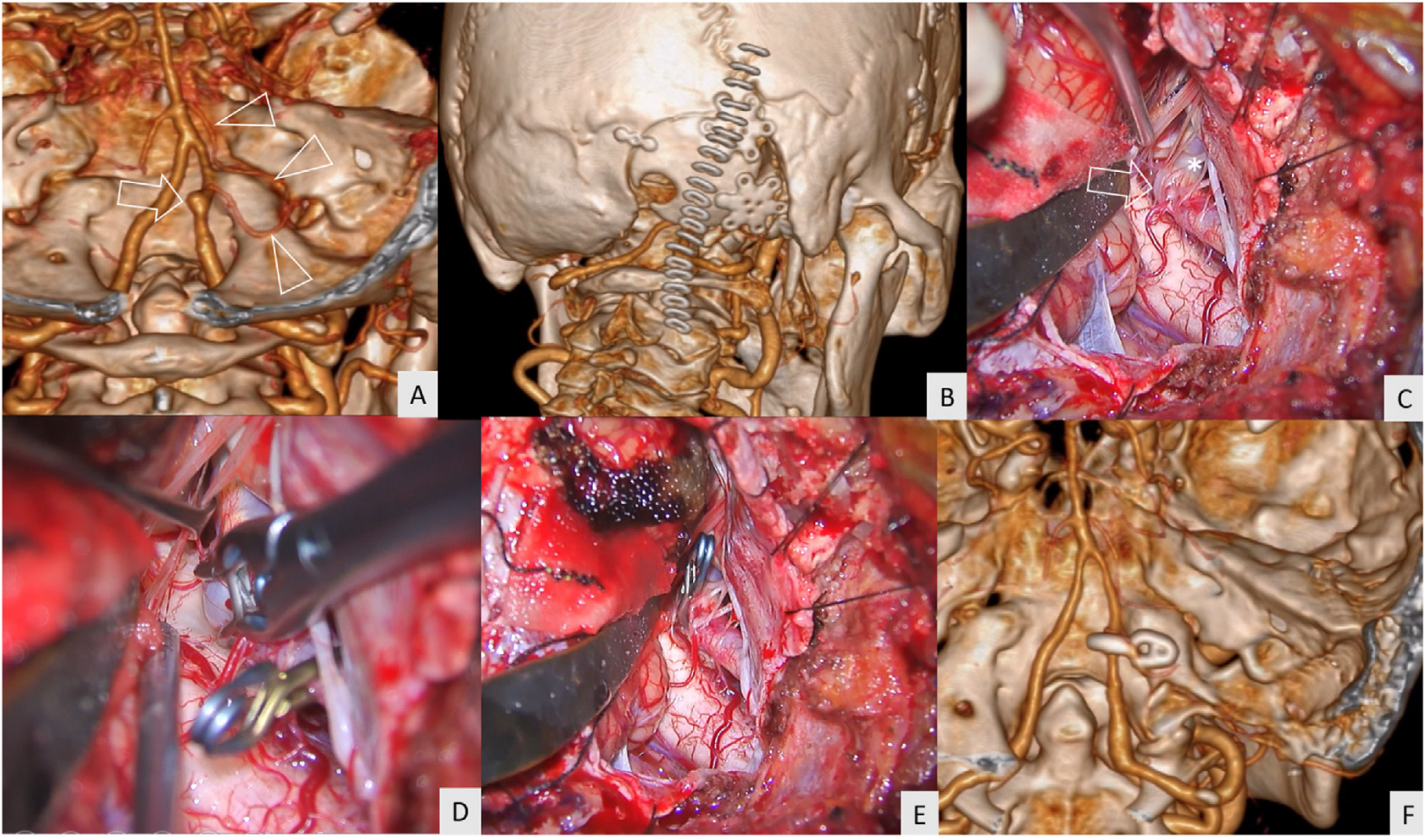
Illustrative case 1 (A) Computed tomography angiography (CTA) demonstrated a saccular aneurysm of the right vertebral artery (arrow) located just above the hypoglossal canal. The anterior inferior cerebellar artery–posterior inferior cerebellar artery (AICA–PICA) loop was identified (arrowhead) (B) The linear paramedian incision (staples) was used for the right far-lateral transcondylar approach (C) A saccular aneurysm (arrow) and the hypoglossal nerve (asterisk) were identified through the right far-lateral transcondylar approach (D) The aneurysm clip was applied to the aneurysm neck (E) Operative field after aneurysm clipping (F) Postoperative CTA revealed complete aneurysm obliteration and good patency of the right vertebral artery.

An 84-year-old female patient presented with incidental finding of a right VA aneurysm. A 4-mm saccular aneurysm of the right VA without PICA involvement was detected by computed tomography angiography (CTA) (Fig. 2A). A right anterior inferior cerebellar artery (AICA)–PICA loop was found. The aneurysm was located just above the hypoglossal canal. The right far-lateral transcondylar approach (Fig. 2B and C) and aneurysm clipping were performed with the standard curve clip (Fig. 2D and E). Complete obliteration of the aneurysm with good patency of the VA was confirmed by CTA immediately after the surgery (Fig. 2F). No further neurological deficits were detected. The patient achieved a GOS of grade 5 at discharge date and 3 months after the surgery (Video 2).

### 4.2 Illustrative case 2

**Fig. 3:**
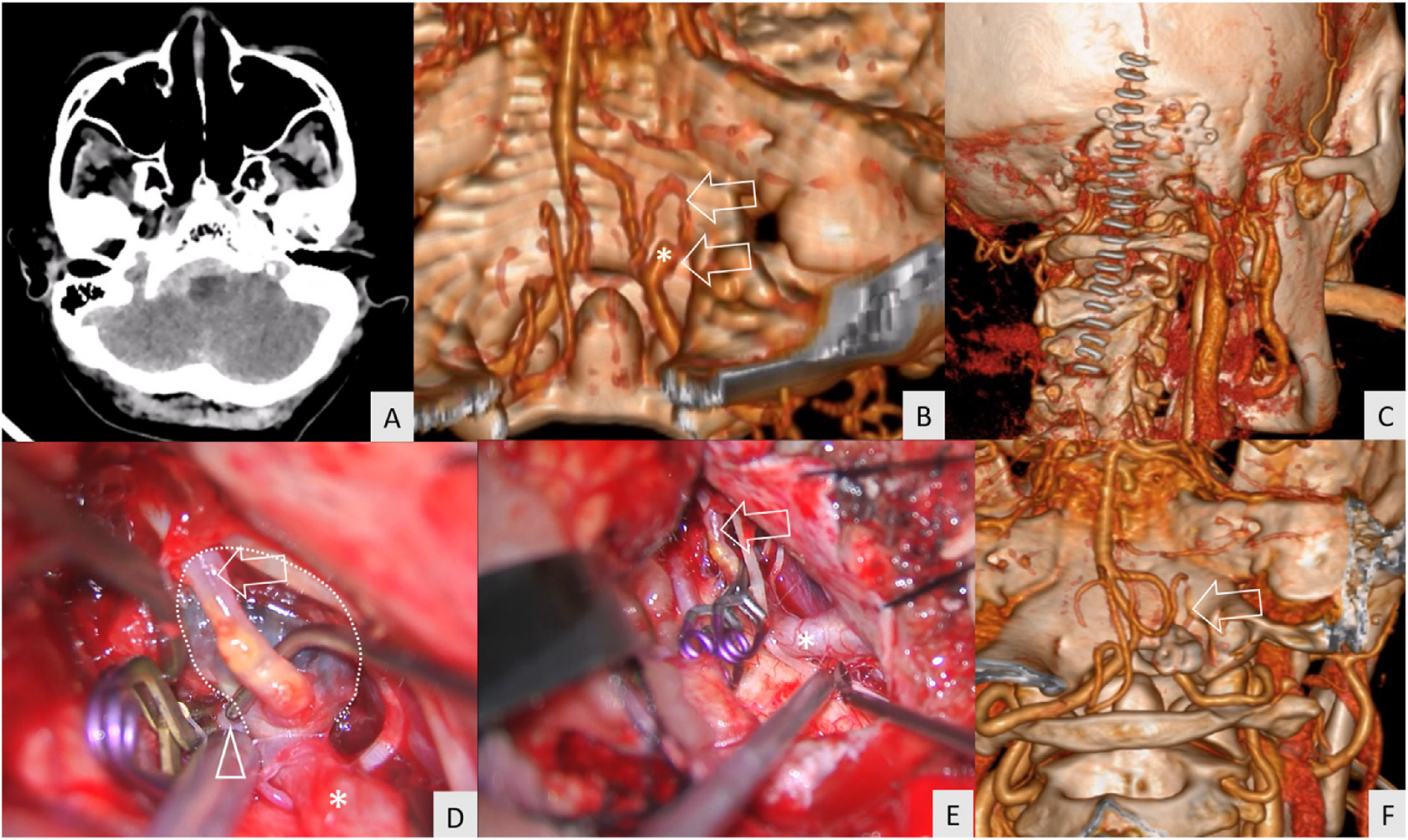
Illustrative case 2 (A) Computed tomography revealed thick subarachnoid clot at the right cerebellomedullary cistern (B) Computed tomography angiography (CTA) demonstrated a saccular aneurysm of the right vertebral artery–posterior inferior cerebellar artery (VA–PICA) junction (asterisk) located below the hypoglossal canal. The arrow shows the course of the posterior inferior cerebellar artery (PICA) (C) The linear paramedian incision (staples) was used for the right far-lateral transcondylar approach (D) A saccular aneurysm (dotted line), PICA (arrow), proximal vertebral artery (asterisk), and distal vertebral artery (arrow head) were identified after proximal and distal control (clip) (E) Aneurysm clips were applied to the aneurysm neck. The PICA (arrow) and proximal vertebral artery (asterisk) were identified after aneurysm clipping (clips) (F) The postoperative CTA revealed complete aneurysm obliteration and good patency of the right vertebral artery and PICA (arrow).

A 63-year-old female patient presented with sudden alteration of consciousness. The WFNS grade was 5 at the first hospital. CT showed a diffuse SAH with predominance in the right cerebellomedullary cistern (Fig. 3A). CTA detected a right VA–PICA aneurysm located below the hypoglossal canal (Fig. 3B). The right far-lateral transcondylar approach (Fig. 3C) and aneurysm clipping were performed (Fig. 3D and E). Complete obliteration of the aneurysm with PICA preservation was confirmed by CTA immediately after the surgery (Fig. 3F). The postoperative course was uneventful. The patient recovered and achieved GOS grade of 3 at 3 months after the surgery (Video 3-1, 3-2).

### 4.3 Illustrative case 3

**Fig. 4:**
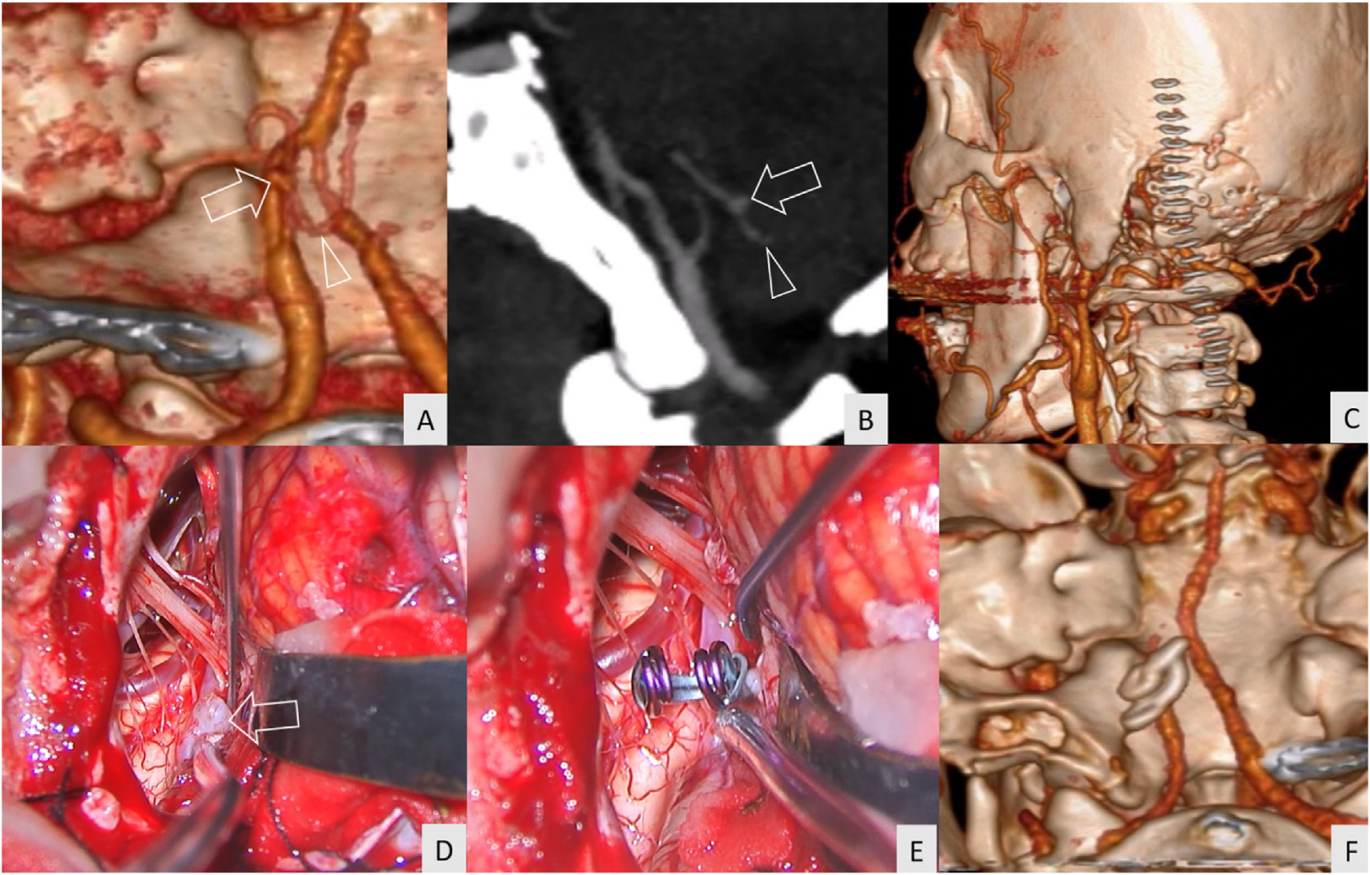
Illustrative case 3 (A, B) Computed tomography angiography (CTA) demonstrated a saccular aneurysm of the left true PICA (arrow) located just proximal to the caudal loop (arrow head) (C) A linear paramedian incision (staples) was used for the left far-lateral transcondylar fossa approach (D) A saccular aneurysm (arrow) was identified through the left far-lateral transcondylar approach (E) The operative field after aneurysm clipping (F) The postoperative CTA revealed complete aneurysm obliteration.

A 39-year-old female patient had an incidental finding of a left true PICA aneurysm located just proximal to the caudal loop (Fig. 4A and B). A linear paramedian incision was used for the left far-lateral transcondylar fossa approach (Fig. 4C). After the aneurysm was identified intraoperatively (Fig. 4D), the aneurysm was occluded while preserving the PICA (Fig. 4E). Complete obliteration of the aneurysm was confirmed by CTA (Fig. 4F). No further neurological deficits were detected. A GOS of 5 was achieved at discharge and 3 months after the surgery (Video 4).

### 4.4 Illustrative case 4

**Fig. 5:**
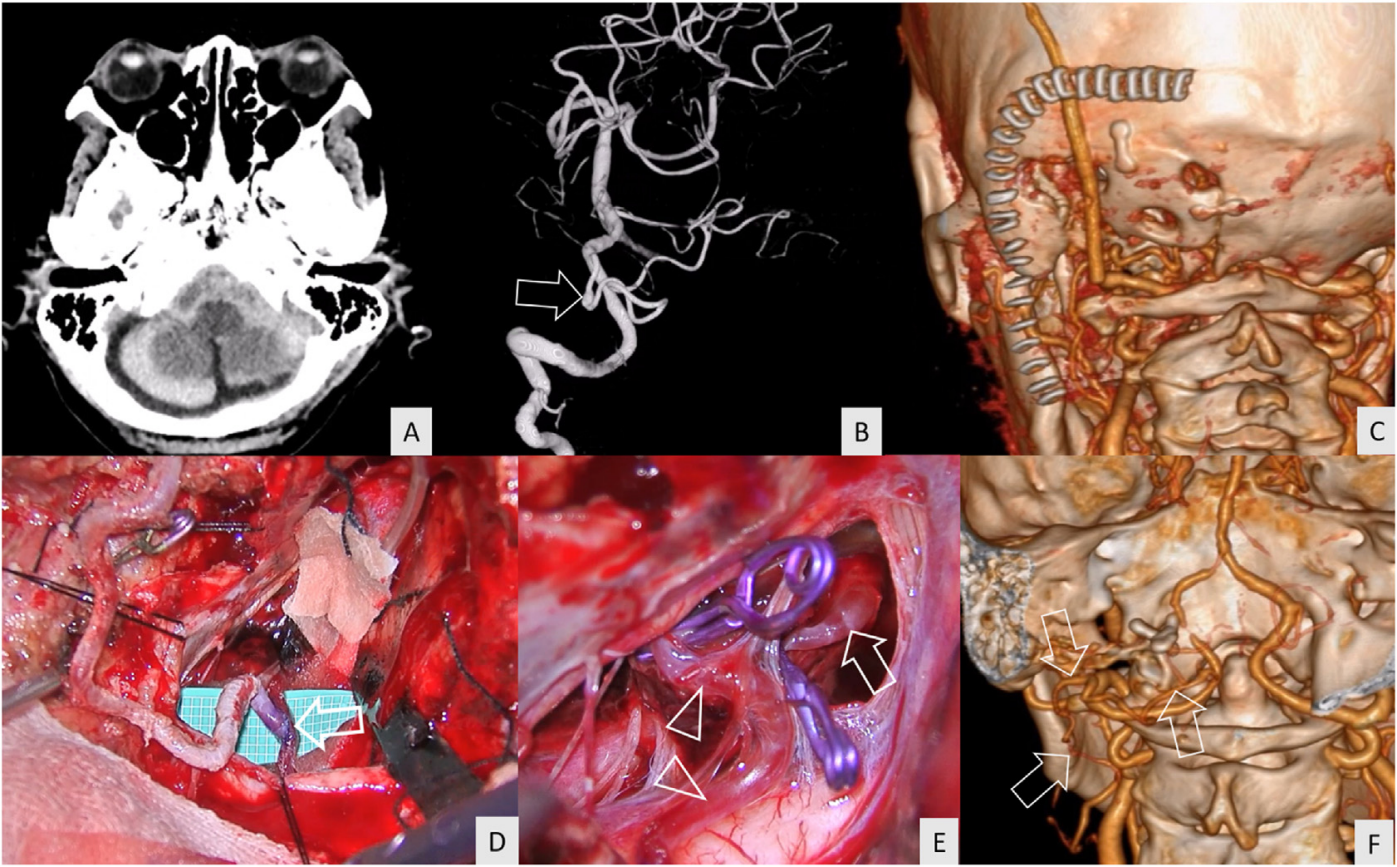
Illustrative case 4 (A) Computed tomography revealed thick subarachnoid hemorrhage at the posterior fossa (B) Digital subtraction angiography demonstrated a fusiform shape aneurysm (arrow) at the lateral medullary segment of the left posterior inferior cerebellar artery (PICA) (C) An “L”-shaped skin incision (staples) was used for occipital artery (OA) harvesting and a left far-lateral transcondylar approach (D) OA–PICA bypass (arrow) was completed (E) The aneurysm was obliterated by complete trapping with the preservation of the proximal (arrow) and distal PICA (arrowhead) (F) Postoperative computed tomography angiography revealed the patency of the OA (arrow).

A 62-year-old female patient presented with a sudden severe headache and initial WFNS grade 2. Posterior fossa SAH was detected by CT (Fig. 5A). A dissecting aneurysm at the lateral medullary segment of the left PICA was demonstrated on digital subtraction angiography (DSA) (Fig. 5B). An “L”-shaped incision was performed for OA harvesting and left far-lateral transcondylar approach (Fig. 5C). OA–PICA bypass (Fig. 5D) and aneurysm trapping (Fig. 5E) were performed. The CTA performed immediately after the surgery showed complete obliteration of the aneurysm with patent OA–PICA bypass (Fig. 5F). No further neurological deficits were detected postoperatively. The patient achieved a GOS of 4 at discharge and 3 months after the surgery (Video 5-1, 5-2).

## 5 Discussion

### 5.1 Endovascular treatment for VA and PICA aneurysms

Endovascular treatment is effective for VA and PICA aneurysms and provides good functional outcomes. Because of the relatively high morbidity and mortality from the complicated surgical approach to the posterior circulation, the treatment of posterior circulation aneurysms was changing from open surgery to endovascular treatment, especially for fusiform or dissecting aneurysms.^1^^,^^3^, ^4^, ^5^^,^^7^

For nondissecting aneurysms, various outcomes were revealed. Spetzler et al reported the 6-year results of 500 patients with ruptured cerebral aneurysms. In total, 21 patients with PICA aneurysms were treated with clipping (*n* = 18) and coiling (*n* = 3). Although the aneurysm obliteration rates were significantly lower and retreatment rates were significantly higher in patients undergoing coiling than in those undergoing clipping, the outcomes for posterior circulation aneurysms continued to favor coiling.^1^ Mericle et al reported 31 patients with proximal PICA aneurysms treated with endovascular techniques. Most patients (84%) presented with SAH with a high percentage of poor grades (88%). Favorable outcome was achieved in 68% with 100% complete aneurysm obliteration.^22^ Chalouhi et al reported 76 patients with PICA aneurysms treated with endovascular techniques. Favorable outcomes were achieved in 93% and 78.7% of the cases in the unruptured and ruptured groups, respectively, but the residual aneurysm rate was 36.6%.^23^ Yin et al reported the 4-year results of 55 patients with PICA aneurysms (*n* = 13 rupture; *n* = 42 unruptured). Endovascular treatment and surgical clipping were performed in 26 and 29 patients, respectively. They found that endovascular treatment achieved better outcomes (modified Rankin scale score ≤2) than surgical treatment (96.2% vs. 89.7%).^24^ Juszkat et al reported the endovascular treatment for 38 patients with ruptured PICA saccular aneurysms. Aneurysms were located at the proximal PICA in 32 patients (84%). Good outcomes were achieved in 60.5% of the patients, with 29.4% recurrent rate.^25^

For dissecting aneurysms, studies have reported treatment results. Liu et al reported 56.7–100% aneurysm obliteration in 196 patients (13% SAH) with VA dissecting aneurysms treated with various endovascular techniques. Favorable outcomes (modified Rankin score 0.5 ± 1.1) were achieved in this series. The most challenging case for endovascular therapy was a ruptured vertebral dissecting aneurysm with PICA involvement.^26^ Catapano et al reported 91 patients with VA dissecting aneurysms treated with endovascular techniques; 32% of the patients had a poor neurological status at presentation. Favorable outcomes were achieved in 68%. The rates of procedural complications (8%) and retreatment (13%) were significantly higher with stent/coil treatment than with vessel sacrifice or flow diversion devices.^2^

### 5.2 Open surgery for VA and PICA aneurysms

For patients with limitations for endovascular treatment, such as tortuosity or atherosclerosis of the VA, allergies to contrast agents, or financial considerations, open surgery was inevitable and should be performed with the appropriate surgical strategy to decrease surgical complications, especially lower cranial nerve deficits.^27^^,^^28^

Many case series of microsurgical treatment with various clinical characteristics and outcomes have been presented. Matsushima et al reported the success of microsurgical treatment of eight patients with VA–PICA saccular aneurysms (six ruptured and two unruptured ones) via seven transcondylar fossa approaches and one transcondylar approach. Transient postoperative lower cranial nerve deficit was displayed in three (37.5%) patients: two for hypoglossal nerve palsy and one for dysphagia.^20^ Al-khayat et al reported 52 patients with VA and PICA aneurysms treated with microsurgery. Favorable outcomes were accomplished in 90.3%, but postoperative lower cranial nerve palsy was high (48%).^29^ Rodríguez-Hernández et al reported 51 (64% SAH) patients with PICA aneurysms treated with various microsurgical techniques via the far-lateral approach. Proximal PICA aneurysms (located inside the vagoaccessory triangle) were found in 43 (84%) patients. Good outcomes were achieved in 80% of the patients, with 100% obliteration rate and 10% complication rate.^12^

Spetzler et al reported 6-year results of 500 patients with ruptured cerebral aneurysms, of which 21 patients with PICA aneurysms were treated with clipping (*n* = 18) and coiling (*n* = 3). For PICA aneurysms, clipping had a significantly higher incidence of poor outcomes than coiling at 3-year (58.3% vs. 26.9%) and 6-year (62.1% vs. 29.6%) follow-up.^1^ Williamson et al reported the 4-year results of 21 patients with ruptured PICA aneurysms (saccular aneurysms, *n* = 14; fusiform and dissecting aneurysms, *n* = 8) treated with microsurgery (*n* = 19) and endovascular methods (*n* = 2). They found that patients with PICA aneurysms were more likely to have a fusiform aneurysm (36% vs. 12%) and had a higher incidence of lower cranial nerve dysfunction than patients without PICA aneurysms (50% vs. 16%). Patients with PICA aneurysms had a significantly higher incidence of poor outcome than all other patients with aneurysm at discharge. Both the high rates of lower cranial nerve dysfunction and fusiform aneurysms in this cohort may help explain this poor outcome.^30^ Ota et al reported the outcomes of 59 patients with VA aneurysms accessed by the transcondylar fossa approach. They focused on the drainage pattern of the posterior condylar emissary vein, but treatment outcome was not described.^16^ Seoane et al reported 52 patients with proximal PICA aneurysms treated with the far-lateral approach without drilling the occipital condyle. Favorable outcome was accomplished in 92.8%, but postoperative lower cranial nerve palsy was relatively high (25%).^31^ Palanisamy et al reported 20 patients with unruptured VA and VA–PICA aneurysms successfully treated by the transcondylar fossa approach while preserving the C1 lamina. One patient (5%) with wrapping of the aneurysm showed aneurysms of the same size postoperatively. One patient (5%) developed transient postoperative lower cranial nerve deficit, which completely recovered at 6 months postoperatively and one patient suffered from CSF rhinorrhea postoperatively, which needs ventriculoperitoneal shunt. No postoperative clinical outcome was described in this study.^12^ Fatehi et al reported 50 (70% rupture) patients with PICA aneurysms treated with microsurgery (*n* = 39) and endovascular therapy (*n* = 11). Residual neck or residual aneurysms were detected in 4 (10.2%) patients treated with microsurgery. They concluded no significant difference in outcomes between the microsurgical and endovascular treatment groups at short- and long-term follow-ups.^32^ Del Maestro et al reported 25 (60% rupture) patients with PICA aneurysms treated with microsurgery (92% by direct clipping and 8% by PICA–PICA bypass with trapping) via the far-lateral approach, and 60% of the patients achieved good outcomes.^33^

For dissecting aneurysms, three surgical series were found. Frisoli et al reported 42 patients with vertebrobasilar dissecting aneurysms treated with various microsurgical techniques. Of these patients, 34 (81%) presented with SAH and 38% of this group was classified to have poor clinical grade at presentation. The complete aneurysm obliteration rate was 95%, and the surgical complication rate was 7%. Good outcomes were observed in 20 (48%) of the 42 patients.^9^ Wongsuriyanan et al reported seven patients with ruptured unclippable VA aneurysms (include dissecting aneurysms) with PICA involvement (71.4% had poor grades) treated with OA–PICA bypass and blind-alley formation (clip occlusion of the proximal VA and PICA origin). The complete obliteration rate of aneurysms was 100%, and favorable outcomes were achieved in 42.9% of the cases 1 month after the surgery. OA–PICA bypass was patent in all patients, and no PICA infarction was detected in this series.^21^ Durongwatana et al reported 22 patients with VA dissection (91% rupture) treated with pure microsurgery. Four dissections types (PICA, pre-PICA, post-PICA, and non-PICA type) were employed with various methods according to their proposed treatment algorithm. Complete obliteration was accomplished in 100%, and favorable outcomes were achieved in 86.4 and 100% for the overall and good-grade groups, respectively.^13^

### 5.3 Open surgery and endovascular treatment for VA and PICA aneurysms

For saccular PICA aneurysms, a meta-analysis demonstrates that microsurgical clipping results in superior angiographic outcomes, comparable functional outcomes, but higher rates of lower cranial nerve palsy than endovascular treatment.^7^

For dissecting aneurysms of the VA and proximal PICA, two meta-analyses have demonstrated that endovascular treatment was associated with high rates of complete occlusion, good long-term neurologic outcomes, and low recurrence and mortality rates. Deconstructive techniques are associated with higher occlusion rates. PICA involvement may lower the efficacy of the endovascular treatment.^26^^,^^34^^,^^35^ With open surgery, high occlusion rates and good outcomes can be achieved by an appropriate surgical strategy and adequate skull-based approach. Appropriate PICA revascularization and optimal clip occlusion were needed for dissecting aneurysms with PICA involvement with high rates of favorable outcomes.^13^^,^^21^ No meta-analysis compared the effectiveness and safety of open surgery and endovascular treatment for dissecting aneurysms.

In the present study, we collected 48 patients with VA and proximal PICA aneurysms (including saccular, dissecting, and fusiform aneurysms) treated with pure microsurgical techniques via the far-lateral approach. The outcomes of this study were comparable with those of a large case series (*n* ≥ 30) of microsurgery and endovascular treatment (Table 4).^2^^,^^8^, ^9^, ^10^, ^11^^,^^22^^,^^23^^,^^25^^,^^26^^,^^29^^,^^31^

**Table 4:** Treatment outcomes of the large clinical studies involving vertebral artery and posterior inferior cerebellar artery aneurysms.

Series (year)	Treatment	No. Of patients	SAH	Poor grade	Type of aneurysms	Overall favorable outcomes	Residual aneurysms	New deficits/procedural complications
Al-khayat et al^29^ (2005)	**Open surgery**	52	65%	53%	PICA aneurysm, *n* = 47 VA aneurysm, *n* = 5	90.3	NA	48%
Mericle et al^22^ (2006)	Endovascular technique	31	84%	88%	Proximal PICA	68%	0%	10%
Rodriguez-Hernandez et al^11^ (2011)	**Open surgery**	43 (proximal PICA)	64% (all PICA)	38% (all PICA)	PICA aneurysm	80% (all PICA)	0%	10% (all PICA)
Chalouhi et al^23^ (2013)	Endovascular technique	76	80%	47%	PICA aneurysms -Dissecting, *n* = 9 -Saccular, *n* = 67	Unruptured, 93% Ruptured, 78.7%	36.6%	12.7%
Lehto et al^8^ (2014)	**Open surgery**	30 (proximal PICA)	93% (all PICA)	62% (all PICA)	Saccular 57% Fusiform 43%	69% (all PICA)	Saccular, 6% Fusiform, 12% (all PICA)	19% (all PICA)
Bohnstedt et al^10^ (2015)	**Open surgery** and endovascular technique	79 (VA ​+ ​proximal PICA)	78% (VA ​+ ​proximal PICA)	NA	Open surgery: VA and proximal PICA, *n* = 50 (63.3%) -Saccular, *n* = 40 (80%) -Dissecting, *n* = 2 (4%) -Fusiform, *n* = 8 (16%) Endovascular: VA and proximal PICA, *n* = 29 (36.7%) -Saccular, *n* = 22 (76%) -Dissecting, *n* = 6 (21%) -Fusiform, *n* = 1 (3%)	NA	Rebleed -Open surgery = 0 -Endo = 1	Open surgery, 22 (44%) Endovascular, 3 (10%)
Juszkat et al^25^ (2016)	Endovascular technique	38	100%	47%	Proximal PICA saccular, *n* = 32 (84%)	60.5% (all PICA)	Recurrent 29.4% (all PICA)	4 (10%)
Seoane et al^31^ (2017)	**Open surgery**	56 (proximal ​+ ​distal PICA)	60.7%	NA	Proximal PICA, *n* = 52 (93%)	92.8% (all PICA)	0% (all PICA)	25%
Frisoli et al^9^ (2021)	**Open surgery**	42	63%	NA	Vertebrobasilar dissecting aneurysm	48%	5%	7%
Catapano et al^2^ (2022)	Endovascular technique	91	59%	44%	VA dissecting aneurysm	68%	13%	18%
Liu et al^26^ (2022)	Endovascular technique	196	13%	NA	VA dissecting aneurysm	NA	0–43%	NA
The current study	**Open surgery**	48	87.5	41.7%	VA and proximal PICA: -Dissecting aneurysm, *n* = 28 (58.3%) -Saccular aneurysm, *n* = 19 (39.6%) -Fusiform aneurysm, *n* = 1 (2.1%)	77.1%	0%	8%

Endo, endovascular treatment; NA, not available; PICA, posterior inferior cerebellar artery; SAH, subarachnoid hemorrhage; VA, vertebral artery.

To the best of our knowledge, this study presents a large series of patients with VA and proximal PICA aneurysms with the highest percentage of SAH treated by pure microsurgery using the far-lateral approach in a single institute.

### 5.4 Far-lateral approach and variants

Many variations of the posterolateral skull-based approach to VA and PICA aneurysms were developed (Table 5). In 1986, Heros^36^ described the *modified lateral suboccipital approach* (extreme-lateral removal of the foramen magnum rim toward the condylar fossa, just posterior to the occipital condyle, with C1 laminectomy) for treating VA and proximal basilar trunk aneurysms without resecting the condyle. In 1990, Sen and Sekhar^37^ described the *extreme-lateral approach* to intradural lesions of the cervical spine and foramen magnum. This approach was the same as the modified lateral suboccipital approach described by Heros^36^ but added the removal of the posterior part of occipital condyle and lateral mass of C1. In 1991, Bertalanffy et al^38^ described the *transcondylar approach,* which is same as the extreme-lateral approach described by Sen and Sekhar,^37^ but added the removal of the jugular tubercle.

**Table 5:** Posterolateral approach to the anterolateral foramen magnum and extent of bone resection.

Extent of bone resection	Posterolateral rim of the foramen magnum	Posterior arch of C1	Posterior part of the occipital condyle	Jugular tubercle
Modified lateral suboccipital approach (Heros^36^ 1986)	Y	Y	*N*	*N*
Extreme lateral approach (Sen and Sekhar^37^ 1990)	Y	Y	Y	*N*
Transcondylar approach (Bertalanffy et al^38^ 1991)	Y	Y	Y	Y
Extreme-lateral inferior transtubercular exposure (ELITE) (Day et al^40^ 1997)	Y	Y/*N*	Y	Y
Transcondylar fossa approach (Matsushima et al^41^ 1998)	Y	Y/*N*	*N*	Y
Far-lateral approach without drilling the occipital condyle (Seoane et al^31^ 2017)	Y	Y	*N*	*N*
Modified transcondylar fossa approach (Palanisamy et al^12^ 2018)	Y	*N*	*N*	Y
This study	Y	*N*	Y/*N*	Y

*N*, no; Y, yes.

With the extreme-lateral approach and transcondylar approach, the atlanto-occipital joint is partially damaged, leading to instability and postoperative cervical pain.^28^^,^^39^ In 1997, Day et al^40^ described the *extreme-lateral inferior transtubercular exposure (ELITE),* which involved the removal of the anterolateral rim of the foramen magnum, jugular tubercle, and posterior one-third of the occipital condyle. The lateral portion of the C1 posterior arch was removed in selected cases that require more inferior exposure. They emphasized that the removal of the anterolateral rim of the foramen magnum and jugular tubercle is the key step for the anterior exposure of the lower brainstem. In 1998, Matsushima et al^41^ described the *transcondylar fossa (supracondylar transjugular tubercle) approach* to the foramen magnum. This approach is almost the same as the transcondylar approach described by Bertalanffy et al^38^; however, the occipital condyle and atlanto-occipital joint were preserved. With this approach, the condylar fossa is removed laterally to the sigmoid sinus and anteriorly to the hypoglossal canal. The posterior part of the jugular tubercle above the hypoglossal canal is also drilled away to the anterolateral foramen magnum. The posterior arch of the C1 was added when the inferior approach was required. This approach is suitable for the lesion located above the hypoglossal canal. In 2017, Seoane et al^31^ reported the *far-lateral approach without drilling the occipital condyle* to treat VA–PICA aneurysms. The lateral rim of the foramen magnum was removed with C1 hemilaminectomy. The condylar fossa and occipital condyle were preserved. This approach was the same as the modified lateral suboccipital approach described by Heros.^36^ In 2018, Palanisamy et al^12^ reported the *modified transcondylar fossa approach* (transcondylar fossa approach while preserving the C1 lamina) for the treatment of VA and VA–PICA aneurysms.

Matsushima et al^20^ described the important anatomical landmarks for the transcondylar fossa approach. The landmarks of the condylar fossa were the 1) posterior condylar canal and emissary vein in the canal and 2) sigmoid magnum triangle which is a part of the condylar fossa. The condylar fossa is the posterior portion of the jugular tubercle, and the tip of the sigmoid magnum triangle is the jugular tubercle.^42^ If the lesion is located below the hypoglossal canal, the medial parts of the occipital condyle and lateral mass of C1 are removed in addition to the transcondylar fossa approach described above (transcondylar approach).^20^

This study reported the successful far-lateral approach without C1 laminectomy to access VA and proximal PICA aneurysms located above the lower border of the foramen magnum. The condylar fossa was drilled in all cases (transcondylar fossa approach). The drilling of the posteromedial part of the occipital condyle was added (transcondylar approach) when the aneurysm is located at or below the level of the hypoglossal canal. According to the results of this study, C1 laminectomy was not necessary for VA and proximal PICA aneurysms located above the lower border of the foramen magnum. When the aneurysm is located below the foramen magnum, C1 laminectomy should be added to the transcondylar approach.

### 5.5 Limitations of the current study

This study has some limitations. First, it was a retrospective descriptive study. Second, postoperative outcomes depend on many factors, including aneurysm characteristics (saccular or dissecting type), aneurysm location (PICA or non-PICA involvement), and preoperative clinical status of the patients (particularly in ruptured cases). Third, the follow-up period was relatively short because most patients were referred from a hospital far from our institute. Finally, CTA was routinely used for preoperative and postoperative vascular imaging, which provides lower-quality images of vascular structures, compared with DSA.

## 6 Conclusions

Microsurgery is a safe and effective treatment of VA and proximal PICA aneurysms. The far-lateral transcondylar or transcondylar fossa approach without C1 laminectomy was adequate and effective for aneurysms located above the lower border of the foramen magnum.

## CRediT authorship contribution statement

**Kitiporn Sriamornrattanakul:** Writing – review & editing, Writing – original draft, Methodology, Investigation, Formal analysis, Conceptualization. **Nasaeng Akharathammachote:** Supervision, Methodology. **Areeporn Chonhenchob:** Supervision, Conceptualization. **Atithep Mongkolratnan:** Investigation, Conceptualization. **Nattawut Niljianskul:** Visualization, Conceptualization. **I-sorn Phoominaonin:** Investigation, Conceptualization. **Chanon Ariyaprakai:** Investigation, Conceptualization.

## Declaration of competing interest

The authors declare that they have no known competing financial interests or personal relationships that could have appeared to influence the work reported in this paper.

## Abbreviations

AICA: anterior inferior cerebellar artery
CTA: computed tomography angiography
GOS: Glasgow outcome score
OA: occipital artery
PICA: posterior inferior cerebellar artery
SAH: subarachnoid hemorrhage
VA: vertebral artery
WFNS: World Federation of Neurosurgical Societies
